# Supplementary material for: Time‑resolved multi-omic analysis of paclitaxel exposure in human iPSC‑derived sensory neurons unveils mechanisms of chemotherapy‑induced peripheral neuropathy
Source: Cell Death Dis. 2026 Feb 10;17(1):211. doi: 10.1038/s41419-026-08445-2 (PMC12921266; doi:10.1038/s41419-026-08445-2)
Supplement: Supplementary file 15 — Dataset 1. [file 41419_2026_8445_MOESM15_ESM.zip › CDD_Open_Data/04_RNA_Seq/Timeline_panel_plots_etc_2023-01-23_1609.zip!/Panel_Plots_etc/Evidence_Plots/post5d/evidence_plot_standard contrasts_msigdb_hallmark_5d_PTX_vs_DMSO_ID6_M5924_HALLMARK_MTORC1_SIGNALING.pdf]

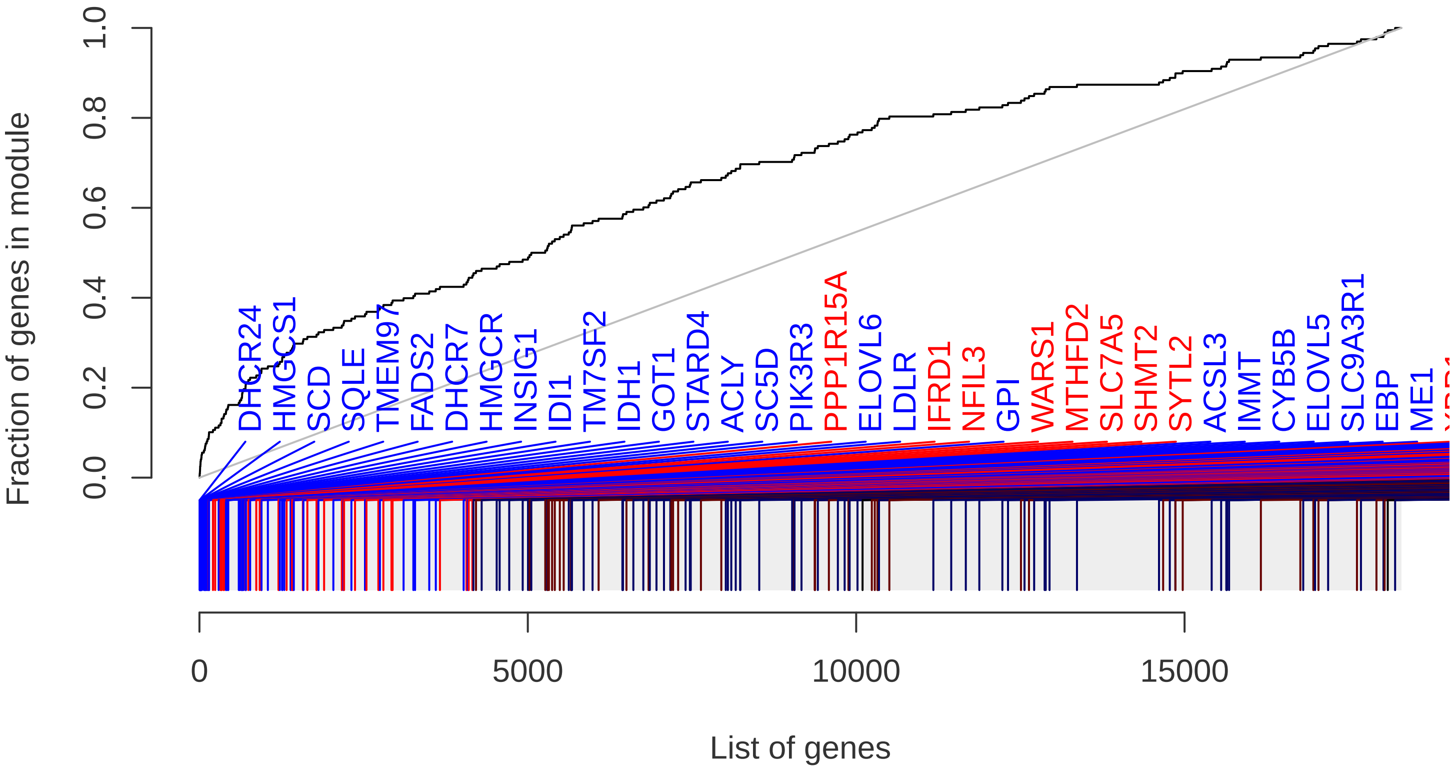

Fraction of genes in module
0.0
0.2
0.4
0.6
0.8
1.0
DHCR24
HMGCS1
SCD
SQLE
TMEM97
FADS2
DHCR7
HMGCR
INSIG1
IDI1
TM7SF2
IDH1
GOT1
STARD4
ACLY
SC5D
PIK3R3
PPP1R15A
ELOVL6
LDLR
IFRD1
NFIL3
GPI
WARS1
MTHFD2
SLC7A5
SHMT2
SYTL2
ACSL3
IMMT
CYB5B
ELOVL5
SLC9A3R1
EBP
ME1
0
5000
10000
15000
List of genes
